# Supplementary material for: Splice-Junction-Based Mapping of Alternative Isoforms in the Human Proteome
Source: Cell Rep. Author manuscript; Available in PMC 2020 Jan 15. (PMC6961840; doi:10.1016/j.celrep.2019.11.026)
Supplement: 2 [file NIHMS1546469-supplement-2.zip › DF1/PXD010154/Heart/Heart_50_TTN_IEEPPPAKVPEEPKR.pdf]

A

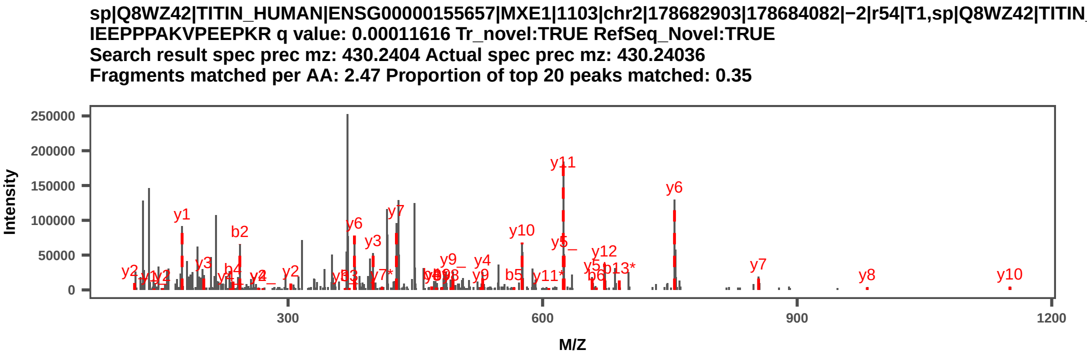
sp|Q8WZ42|TITIN_HUMAN|ENSG00000155657|MXE1|1103|chr2|178682903|178684082|−2|r54|T1,sp|Q8WZ42|TITIN_
IEEPPPAKVPEEPKR q value: 0.00011616 Tr_novel:TRUE RefSeq_Novel:TRUE
Search result spec prec mz: 430.2404 Actual spec prec mz: 430.24036
Fragments matched per AA: 2.47 Proportion of top 20 peaks matched: 0.35
Intensity
250000
200000
150000
100000
50000
0
300
600
900
1200
M/Z
y1
b2
y6
y7
y3
y10
y11
y6
y12
y7
y8
y10

B

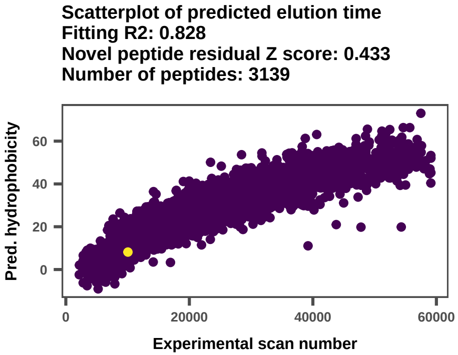
Scatterplot of predicted elution time
Fitting R2: 0.828
Novel peptide residual Z score: 0.433
Number of peptides: 3139
Pred. hydrophobicity
60
40
20
0
0
20000
40000
60000
Experimental scan number

C

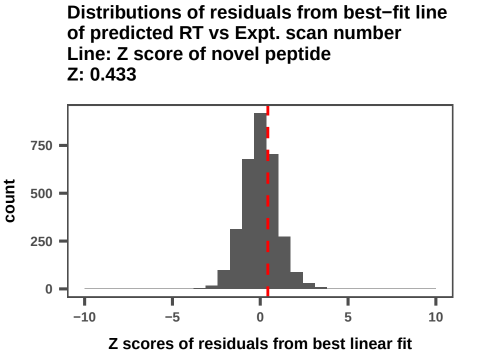
Distributions of residuals from best−fit line
of predicted RT vs Expt. scan number
Line: Z score of novel peptide
Z: 0.433
count
750
500
250
0
−10
−5
0
5
10
Z scores of residuals from best linear fit
